# CD4^+^CD25^high^CD127^-^ regulatory T-cells in COPD: smoke and drugs effect

**DOI:** 10.1186/s40413-016-0095-2

**Published:** 2016-02-12

**Authors:** Alessandra Chiappori, Chiara Folli, Francesco Balbi, Emanuela Caci, Anna Maria Riccio, Laura De Ferrari, Giovanni Melioli, Fulvio Braido, Giorgio Walter Canonica

**Affiliations:** Allergy and Respiratory Diseases, IRCCS San Martino-IST-University of Genoa, Genoa, Italy; Istituto Giannina Gaslini, Genoa, Italy

**Keywords:** Chronic obstructive pulmonary disease (COPD), Inflammation, Regulatory T-cells, Corticosteroids, Budesonide, LABA, Formoterol

## Abstract

**Background:**

Chronic obstructive pulmonary disease (COPD) is a progressive lung disorder characterized by poorly reversible airway obstruction and its pathogenesis remains largely misunderstood. Local changes of regulatory T-cell populations in the lungs of COPD patients have been demonstrated although data concerning their pathologic role are contrasting. The aim of our study was to evaluate the relative percentage of regulatory T-cells in the peripheral blood of current and former smoker subjects, affected or not by COPD. Furthermore, the effect of different concentrations of budesonide and formoterol, on regulatory T-cells has been investigated.

**Methods:**

T regulatory lymphocytes were isolated and assessed as CD4^+^CD25^high^CD127^-^ cells by flow cytometry and cultured for 48 hours in the absence or in the presence of budesonide and/or formoterol at different doses.

**Results:**

CD4^+^CD25^high^CD127^-^ regulatory T-cells percentage was significantly reduced in COPD patients, both current and former smokers, with respect to volunteers. Furthermore, CD4^+^CD25^high^CD127^-^ cells of COPD patients showed a not statistically significant response to drugs compared to healthy subjects.

**Discussion:**

Our results evidenced a different behaviour of CD4^+^CD25^high^CD127^-^ Treg cells in COPD patients after in vitro treatments.

**Conclusions:**

Based on our data, we suggested a possible role of CD4 CD25^high^CD127 T-cells in COPD pathogenesis.

## Background

Chronic obstructive pulmonary disease (COPD) is a progressive lung disorder characterized by poorly reversible airway obstruction. Tobacco smoking is the main etiological factor inducing oxidative stress and an abnormal inflammatory response leading to mucociliary dysfunction, airway wall thickening and pulmonary parenchymal changes [1]. COPD pathogenesis remains largely unknown and it appears to be the result of smoke exposure and host/defense interaction. In balancing the efficient recognition of pathogens and the control of immune tolerance, regulatory T-cells (Tregs) play a key role. Different subtypes of Tregs exist. While the forkhead box P3 transcription factor (FOXP3) is the hallmark of regulatory function, interleukin (IL)-2 receptor α-chain (also known as CD25) is a cell surface marker commonly used to distinguish among regulatory (CD25^high^), activated (CD25^int^), and naive (CD25^low^) T-cells in humans [2]. Liu et al. have demonstrated that the downregulation of the α-chain of the IL-7 receptor (CD127) on the majority of the CD4^+^FOXP3^+^ T-cells distinguishes Tregs from activated T-cells. Low CD127 expression, combined with high expression of CD25, therefore enables better isolation and purification of Treg populations among CD4^+^CD25^+^ T-cells. In functional assays, CD4^+^CD25^high^CD127^low^ T-cells are highly suppressive [3].

Contrasting evidences have been reported concerning different subtypes of CD4^+^FOXP3^+^ T-cells in COPD. Plumb et al. assessed the presence of CD4^+^FOXP3^+^ Tregs in surgically resected lung tissues from COPD patients, smokers with normal lung function and healthy non smokers showing an increased number of CD4^+^FOXP3^+^ cells in lymphocyte follicles in lung parenchyma of moderate COPD patients [4]. Roos-Engstrand et al., analyzing the bronchoalveolar lavage fluid (BALF), showed no significant differences in CD4^+^CD25^+^ cells between COPD patients and the other healthy smokers and non smokers subjects. Among CD4^+^ T-cells expressing CD25, smokers with normal lung function had significantly decreased percentage of FOXP3 expression compared with those who never smoked. Moreover, the authors found that ex-smokers COPD patients expressed a decreased percentage of CD127^+^ cells in BALF compared to smoking COPD patients and the expression of CD127 on CD4^+^CD25^+^ T-cells was increased in smokers with normal lung function, with respect to non-smokers [5]. Compared with never smokers, smokers with normal respiratory function presented a greater number of regulatory T-cells, absent in COPD subjects [6]. Further, an increased proportion of Tregs in the BALF was found in smokers with COPD compared to the control group [7]. Recently, Lane et al. have found that smokers without COPD have increased numbers of CD4^+^CD25^+^FOXP3^+^ Tregs in the large airways [8]. Besides, another study demonstrated impaired Treg-mediated suppression of CD4^+^ T-cell activation in a group of COPD patients with high body mass index and similar proportions of CD4^+^FOXP3^+^ T-cells in COPD patients compared to controls [9].

Tregs have also been explored in peripheral blood. Xiong et al. showed that CD4^+^CD25^+^, CD4^+^ Treg, CD8^+^CD25^+^ and CD8^+^ Tregs were expressed in the peripheral blood of patients with acute exacerbations of COPD with a significant correlation with age, disease’s course, smoking index, quantity of white cells, and blood pH, while no correlations were found between these cells and IL-10 [10]. Barcelò et al. showed no significant differences in peripheral blood samples among healthy smokers, no-smokers and COPD patients, concerning CD4^+^CD25^+^ T-cells [6].

Overall, these data underline a not well understood role of Treg population in the pathogenesis of COPD and further investigations are needed to evaluate the potential effect of drugs on Tregs. Bronchodilators, such as long-acting β_2_-agonists, and inhaled corticosteroids, used in combination, are the recommended treatment for moderate and severe COPD patients with frequent exacerbations. It has been demostrated that glucocorticoids are able to restore the balance between inflammatory and regulatory cells, increasing the proportion of FOXP3^+^ Treg cells [11, 12] but, to date, not many studies assessed the effects of ß_2_-agonists in combination with corticosteroids on these lymphocytes [13].

The aim of our study was to evaluate the relative percentage of CD4^+^CD25^high^CD127^-^ Tregs in the peripheral blood of current and former smoker COPD patients and healthy volunteers. Furthermore, the in vitro effect of different concentrations of an inhaled corticosteroid (budesonide) and a long-acting β_2_-agonist (formoterol), alone and combined, in modulating CD4^+^CD25^high^CD127^-^ Tregs cell population has been investigated.

## Methods

### Study subjects

According with the protocol approved by ethical committees of IRCCS-A.O.U. San Martino-IST of Genoa, healthy volunteers current smokers (CSHV) and never-smokers (NSHV), and COPD patients, former smokers (FSC) and current smokers (CSC), were enrolled from November 2012 to December 2013 among the outpatients attending at Allergy and Respiratory Diseases Clinic of Genoa University for a scheduled visit. COPD diagnosis and functional severity were performed according to the Global Initiative for Chronic Obstructive Lung Disease (GOLD) document 2011 revision [14]. Inclusion criteria were age ≥40 years, clinical diagnosis of COPD and symptoms for more than 2 years, forced expiratory volume in the 1^st^ second (FEV_1_)/forced vital capacity (FVC) post-bronchodilator lower than 70 %, FEV_1_ between 50-80 % of normal predicted, smoking history of at least 10 pack years, on treatment with long acting bronchodilators. Patients having history of asthma and/or allergic rhinitis before the age of 40 years, or suffering from cancer, infections, autoimmune diseases and other immune-related diseases were excluded. No patients treated with chemotherapics, immunosuppressors, oral steroids and antibiotics in the 4 weeks before the enrollment, were recruited. CSHV and NSHV were ≥ 40 years of age with normal spirometry according to American Thoracic Society (ATS)/European Respiratory Society (ERS) criteria. Written informed consent was obtained from all participants before study.

### Isolation of peripheral blood mononuclear cells and immunophenotyping

**Fig. 1:**
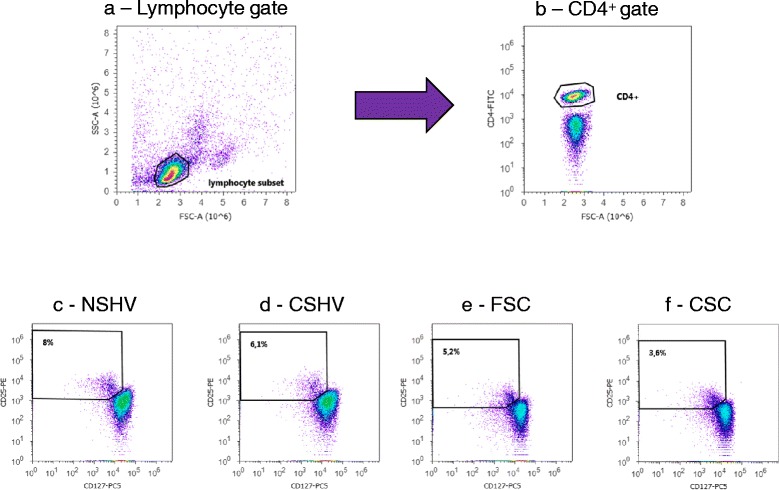
Gating strategy for flow cytometric identification of CD4^+^CD25^high^CD127^-^ regulatory T-cells in the peripheral blood. **a** Lymphocytes were identified based on their characteristic properties shown in the forward scatter (FSC) and sideward scatter (SSC). **b** A representative gating was set for CD4^+^ T cells from blood lymphocytes. **c** A representative dot plots showing expression of CD25^high^CD127^-^ regulatory T-cells in blood CD4^+^ T-cells of a never-smoker healthy volunteer (NSHV). **d** A representative dot plots showing expression of CD25^high^CD127^-^ regulatory T-cells in blood CD4^+^ T-cells of a current smoker healthy volunteer (CSHV). **e** A representative dot plots showing expression of CD25^high^CD127^-^ regulatory T-cells in blood CD4^+^ T cells of a former smoker COPD patient (FSC). **f** A representative dot plots showing expression of CD25^high^CD127^-^ regulatory T-cells in blood CD4^+^ T-cells of a current smoker COPD patient (CSC)

Peripheral blood mononuclear cells (PBMCs) were isolated from the peripheral blood of COPD patients, CSHV and NSHV by means of a density gradient centrifugation (Lympholyte; Cedarlane, Burlington, USA). PBMCs were suspended in RPMI 1640 cell culture medium (Euroclone S.p.A.; Pero, Milan, Italy) and viable cell counts obtained. Regulatory lymphocytes were stained and assessed as CD4^+^CD25^high^CD127^-^ cells. Their percentage as a proportion of the total CD4^+^ cells was tested by flow cytometry before treatments (time t0). The following mAbs were used: CD4-FITC, CD25-PE and CD127-PC5 (Immunotech; Beckman Coulter, Marseille, France). Tregs CD4^+^CD25^high^CD127^-^ were gated from CD4^+^ T-cells (Fig. 1). 100.000 events for each sample were acquired using the Attune Acoustic Focusing Cytometer (Life Technologies, Carlsbad, USA) and the analysis was performed with Attune Cytometric Software 2.1. The results were expressed as percentage of gated CD4^+^ cells.

### Cell culture and drug treatment

We analyzed CD4^+^CD25^high^CD127^-^ cells population by flow cytometry using an incubation time of 48 hours according with previously published experiments [15]. Fresh PBMCs were cultured in 24-well round-bottomed microtiter plate at 2X10^6^ cells/well in RPMI 1640 (Euroclone S.p.A.; Pero, Milan, Italy) supplemented with 10 % heat-inactivated Human AB Serum (Euroclone S.p.A.; Pero, Milan, Italy), 1 % penicillin/streptomycin (Euroclone S.p.A.; Pero, Milan, Italy) and 2.5 mg/l amphotericin B (Sigma-Aldrich; St. Louis; USA). Cells were treated with IL-2 (100 I.U./ml) (Miltenyi Biotec; Auburn, USA) + transforming growth factor beta-1 (TGF-β1) (5 ng/ml) (PeproTech EC Ltd.; London, UK) (NT) and in the absence or in the presence of different dosages of budesonide (Bud) and formoterol (For) either alone or in combination (Bud 1 and 0.01 μM, For 30 and 0.3 nM and Bud 1 μM + For 30 nM and Bud 0.01 μM + For 0.3 nM) (Astrazeneca; Basiglio, Italy) [16, 17]. Cell viability was evaluated by trypan blue exclusion dye assay to rule out drugs toxicity. Drugs concentrations have been chosen considering dose–response experiments from our previous study on NK cells population in COPD patients [15]. Following drugs stimulation, cells were harvested, resuspended in PBS, stained with antibodies and analyzed by flow cytometry (time t1) as above described. In order to exclude a drug solvent effect on cells, the effect of the maximum EtOH dose used to dissolve the drugs was also evaluated. We analyzed CD4^+^CD25^high^CD127^-^ cells population by flow cytometry.

### Population sample and data analysis

Population sample was estimated according with available literature and study power calculation. The Kolmogorov-Smirnov test was applied for assessing the normality of the data distribution. Spearman's rank correlation coefficient was applied to test the correlation between CD4^+^CD25^high^CD127^-^ Treg cells ratios and FEV_1_ values. For multiple comparisons, one-way analysis of variance (ANOVA) was performed, followed by post hoc Duncan’s test. Statistical significance was defined as a p value below 0.05. Statistical analysis was performed using STATISTICA version 6.0 (StatSoft) and GraphPad Prism version 5.0 (GraphPad Software Inc.).

## Results

### Demographic characteristics of study population

PBMCs were obtained from 28 moderate (14 current smokers and 14 former smokers) COPD patients and 20 healthy volunteers (10 current smokers and 10 never-smoker). Clinical and demographic data of study population are reported in Table 1. The mean ages in the patients groups were statistically significant different than in the control groups. The unequal sex ratio is in line with higher prevalence of COPD in men than in women observed in real life.

**Table 1:** Demographic characteristics

	Never-smoker healthy volunteers	Current smoker healthy volunteers	Former smokers COPD patients	Current smoker COPD patients
n	10	10	14	14
Age (years)	61.4	57.8	72.5 §§,***	69.9 §,**
Sex (F/M)	6/4	1/9	1/13	3/11
FEV_1_ (%predicted)	102.6 ± 8.45	94.6 ± 11.9	61.71 ± 8.2 §§, **	57.21 ± 7.3 §§,**

*COPD* chronic obstructive pulmonary disease; *FEV*_1_ forced expiratory volume in the 1^st^ second

Data are presented as mean ± SD

§ = *p* < 0.05 vs never-smoker healthy volunteers

§§ = *p* < 0.01 vs never-smoker healthy volunteers

** = *p* < 0.01 vs current smoker healthy volunteers

*** = *p* < 0.001 vs current smoker healthy volunteers

### Circulating Tregs in COPD patients and healthy volunteers

**Fig. 2:**
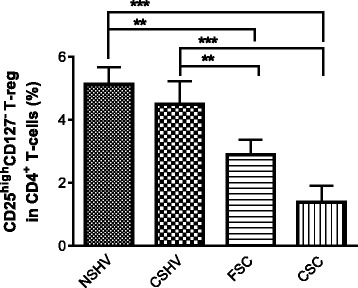
Comparison with the percentages of CD4^+^CD25^high^CD127^-^ regulatory T-cells in the peripheral blood at t0. Data are expressed as mean ± SEM. ** = *p* < 0.01; *** = *p* < 0.001. NSHV: never-smoker healthy volunteers; CSHV: current smokers healthy volunteers; FSC: former smoker COPD patients; CSC: current smoker COPD patients

**Fig. 3:**
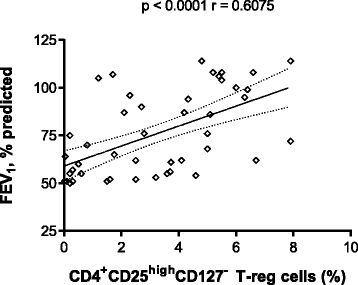
Correlation between CD4^+^CD25^high^CD127^-^ regulatory T-cells ratios and FEV_1_ (%predicted). Linear regression with a 95 % mean prediction interval. FEV_1_: forced expiratory volume in the 1^st^ second

The expression of Treg cells (CD4^+^CD25^high^CD127^-^) in peripheral blood was different among groups (Fig. 2). In particular CD4^+^CD25^high^CD127^-^ percentage was significantly reduced in current smokers COPD patients (CSC) and former smokers COPD patients (FSC) with respect to healthy volunteers never-smokers and current smokers (Fig. 2). Correlating CD4^+^CD25^high^CD127^-^ percentages to FEV_1_ values, we observed a statistically significant correlation (*r* = 0.6075; p < 0.0001), showing that the lower Treg cells are circulating in peripheral blood, the greater will be the FEV_1_ decline (Fig. 3).

### Effect of budesonide and formoterol in cultured PBMCs

**Fig. 4:**
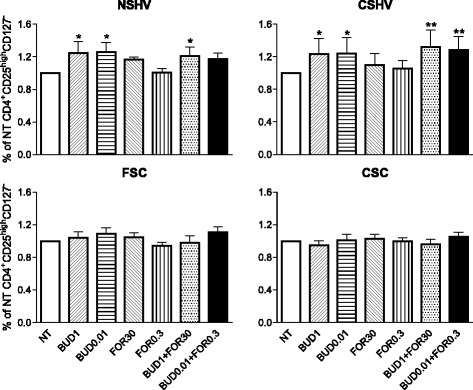
CD4^+^CD25^high^CD127^-^ regulatory T-cells after in vitro treatments. PBMCs from never-smoker healthy volunteers (*n* = 10), current smokers healthy volunteers (*n* = 10), former smoker COPD patients (*n* = 14) and current smoker COPD patients (*n* = 14) were cultured with/without drugs. Data are expressed as mean ± SEM and were normalized to the value of the untreated control. * = *p* < 0.05; ** = *p* < 0.01 versus NT. NT = cells treated with TGF-β + IL-2 alone; BUD1 = cells treated with TGF-β + IL-2 and budesonide 1 μM; BUD0.01 = cells treated with TGF-β + IL-2 and budesonide 0.01 μM; FOR30 = cells treated with TGF-β + IL-2 and formoterol 30 nM; FOR0.3 = cells treated with TGF-β + IL-2 and formoterol 0.3 nM; BUD1 + FOR30 = cells treated with TGF-β + IL-2 and budesonide 1 μM + formoterol 30nM; BUD0.01 + FOR0.3 = cells treated with TGF-β + IL-2 and budesonide 0.01 μM + formoterol 0.3 nM. NSHV: never-smoker healthy volunteers; CSHV: current smokers healthy volunteers; FSC: former smoker COPD patients; CSC: current smoker COPD patients; TGF-β1: transforming growth factor beta-1; IL: interleukin

Statistical analysis of data shows that CD4^+^CD25^high^CD127^-^ cells in COPD patients have not a statistically significant response to budesonide, alone and in combination with formoterol, compared with healthy volunteers. In fact, no treatment significantly modulated the proportion of these cells (Fig. 4). Cell culture supernatants were used to investigate the production of IL-10 by ELISA test (data not shown). There were no differences in IL-10 production between groups (*p* = 0.1051).

No difference with untreated cells was evidenced with the maximum EtOH dose used.

## Discussion

Several mechanisms have been proposed to be involved in the development of COPD: oxidative stress due to tobacco smoking [18], activation of neutrophils and macrophages, apoptosis of endothelial and epithelial cells [19], defective efferocytosis of residual apoptotic debris [20], viral infections [21] and genetic susceptibility [22].

Many of these hypothesis ascribe to environmental factors a central role in the inflammatory response observed in COPD, nonetheless this inflammatory state is a self-perpetuating process able to persist for years after cessation of smoking. For this reason it has been proposed that COPD may derive from a shift from the non-specific innate response present in every smoker toward an adaptive immune response and may present an autoimmune component. Exposure to infections or smoking-induced lung injury could release sequestered autoantigens and DNA from apoptotic cells and alter proteins [23]. T lymphocytes can recognize these products as foreign antigens and maintain a prolonged inflammatory state in the airways in response to self-antigens [24]. Activation of T-cells is highly controlled by negative regulatory mechanisms. Disturbed homeostasis of regulatory T-cell population was demonstrated in several pathologies with autoimmune etiology such as lupus erythematosus, diabetes mellitus and rheumatoid arthritis [25, 26]. A deficiency of regulatory T-cells can weaken the immune tolerance to self-antigens and thereby support a persistent inflammation mediated by CD8^+^ cells in COPD. Lee et al. hypothesized that, in patients with emphysema, the inflammatory process would be sustained by the presence of anti-elastin autoantibodies and showed that the Treg population, detected as CD25^high^CD62L^+^ cells, was lower in the lungs and in the blood of patients compared to healthy subjects [27].

Data concerning regulatory T-cells in COPD patients are not so numerous and sometimes discordant. In our study we investigated CD4^+^CD25^high^CD127^-^ proportion in peripheral blood, in current and former smokers with moderate airway obstruction COPD patients and current smokers and never-smokers healthy volunteers. We found a significant depletion in CD4^+^CD25^high^CD127^-^ proportion in COPD patients compared to healthy smokers and never-smoker subjects. This might reflect a kind of progression of inflammation status and exhaustion of anti-inflammatory responses from healthy smokers to COPD patients. Evaluating Treg population as CD4^+^CD25^high^CD127^-^, we confirmed data obtained in other studies [28, 29]. However, our results are in contrast with the findings of Barcelò et al., showing that no differences in CD4^+^CD25^+^ Tregs from peripheral blood were detected among COPD, healthy smokers and controls and with the results of Vargas-Rojas et al. describing increased levels of Treg cells present in COPD and smokers subjects compared to healthy ones [6, 30]. These differences may be caused by patients selection or technical approach to evidence regulatory T-cells. Moreover, we found statistically significant differences in mean age between groups. Based on literature, peripheral CD4^+^CD25^high^CD127^-^ cells have to increase with patient’s age, but our data support the opposite. Thus, we suppose that these results can be mainly related to COPD pathology [31–34]. Other studies are necessary to increase the number of samples and to definitively clarify the role of regulatory T-cells in COPD.

Hopefully, pharmacological treatment might restore the balance between effector T-cells and regulatory T-cells [35]. Profita et al. evaluated the expression of FOXP3 in PBMCs from COPD patients after 48 h of in vitro stimulation with tiotropium and olodaterol. They reported increased levels of CD4^+^CD25^+^FOXP3^+^ in treated PBMCs with respect to untreated ones with both drugs alone or in combination [36].

In our study, stimulated in vitro COPD patients CD4^+^CD25^high^CD127^-^ cells were not modulated by budesonide or formoterol, both alone and in combination. Interestingly, these cells were significantly modulated by budesonide treatments in never-smoker and current smokers healthy volunteers except by Bud 0.01 μM + For 0.3 nM in NSHV. Yang et al. showed that in patients with moderate or severe COPD receiving treatment with 50/500 μg of salmeterol/fluticasone propionate twice a day for 12 weeks, the proportion of FOXP3^+^ Tregs in the total CD4^+^ T-cell population in the peripheral blood was drastically higher than that before treatment [29]. The different effect of salmeterol/fluticasone and budesonide/formoterol on peripheral Treg cells should be evaluated considering the different methodological approach adopted in the studies.

Nevertheless, if future studies will confirm these results, they should be analyzed bearing in mind the different rate of drug-related adverse events, such as pneumonia, described in clinical research. In fact, among the potential side effects of inhaled corticosteroid (ICS) treatments in COPD patients, the use of fluticasone or fluticasone/salmeterol combination has been associated with a higher prevalence of pneumonia in the major long-term studies [37–39]. All ICSs can suppress natural and adaptive immunity with a potentially greater inhibition of type-1 innate immunity [40]. On the other hand, no similar increased risk of pneumonia has been reported in patients with COPD treated with the budesonide/formoterol combination [41–43].

## Conclusions

Our data pointed out a different behavior of CD4^+^CD25^high^CD127^-^ T-cells in the four groups evaluated, depending on the presence of COPD inflammatory process. In COPD patients, Treg cells appeared unsusceptible to the action of drugs, whose effect is, on the contrary, clear on cellular components of healthy subjects.

In conclusion, we support the possible role of CD4^+^CD25^high^CD127^-^ in COPD pathogenesis. Budesonide and formoterol tested in vitro did not have any effects on CD4^+^CD25^high^CD127^-^ population in our experimental conditions. These results need to further be explored in a direct comparison with other bronchodilators and ICSs in order to better clarify their immunomodulatory properties.

## Abbreviations

COPD: Chronic obstructive pulmonary disease
Bud: Budesonide
For: Formoterol
Tregs: Regulatory T-cells
FOXP3: Forkhead box P3 transcription factor
IL: Interleukin
CD127: α-chain of IL-7 receptor
BALF: Bronchoalveolar lavage fluid
CSHV: Current smoker healthy volunteers
NSHV: Never-smoker healthy volunteers
FSC: Former smoker COPD patients
CSC: Current smoker COPD patients
GOLD: Global initiative for chronic obstructive lung disase
FEV_1_: Forced expiratory volume in the 1^st^ second
FVC: Forced vital capacity
ATS: American Thoracic Society
ERS: European Respiratory Society
PBMC: Peripheral blood mononuclear cell
TGF-β1: Transforming growth factor beta-1
ICS: Inhaled corticosteroid

## References

[1] Hogg JC (2004). Pathophysiology of airflow limitation in chronic obstructive pulmonary disease. Lancet.

[2] Perz JB, Gürel S, Schonland SO, Hegenbart U, Ho AD, Dreger P. CD4^+^CD25^high^CD127^low^ regulatory T cells in peripheral blood are not an independent factor for chronic graft-versus-host disease after allogeneic stem cell transplantation. Scientific World Journal. 2012; doi: 10.1100/2012/606839. 10.1100/2012/606839 PMC3361289 22666141

[3] Liu W, Putnam AL, Xu-Yu Z, Szot GL, Lee MR, Zhu S (2006). CD127 expression inversely correlates with FoxP3 and suppressive function of human CD4^+^ T reg cells. J Exp Med.

[4] Plumb J, Smyth LJ, Adams HR, Vestbo J, Bentley A, Singh SD (2009). Increased T-regulatory cells within lymphocyte follicles in moderate COPD. Eur Respir J.

[5] Roos-Engstrand E, Pourazar J, Behndig AF, Bucht A, Blomberg A (2011). Expansion of CD4^+^CD25^+^ helper T cells without regulatory function in smoking and COPD. Respir Res.

[6] Barceló B, Pons J, Ferrer JM, Sauleda J, Fuster A, Agustí AG (2008). Phenotypic characterization of T-lymphocytes in COPD: abnormal CD4^+^CD25^+^ regulatory T-lymphocyte response to tobacco smoking. Eur Resp J.

[7] Smyth LJ, Starkey C, Vestbo J, Singh D (2007). CD4-regulatory cells in COPD patients. Chest.

[8] Lane N, Robins RA, Corne J, Fairclough L (2010). Regulation in chronic obstructive pulmonary disease: the role of regulatory T-cells and Th17 cells. Clin Sci.

[9] Tan DB, Fernandez S, Price P, French MA, Thompson PJ, Moodley YP (2014). Impaired function of regulatory T-cells in patients with chronic obstructive pulmonary disease (COPD). Immunobiology.

[10] Xiong XZ, Jin Y, Zhou Q, Zhang XJ, Du W, Liu W (2008). Correlation between FoxP3^(+)^ regulatory T cells and chronic obstructive pulmonary disease. Zhonghua Yi Xue Za Zhi.

[11] Karagiannidis C, Akdis M, Holopainen P, Woolley NJ, Hense G, Rückert B (2004). Glucocorticoids upregulate FOXP3 expression and regulatory T cells in asthma. J Allergy Clin Immunol.

[12] Pace E, Di Sano C, La Grutta S, Ferraro M, Albeggiani G, Liotta G (2012). Multiple in vitro and in vivo regulatory effects of budesonide in CD4^+^ T lymphocytes subpopulations of allergic asthmatics. PLoS One.

[13] Peek EJ, Richards DF, Faith A, Lavender P, Lee TH, Corrigan CJ (2005). Interleukin-10-secreting “regulatory” T cells induced by glucocorticoids and β_2_-agonists. Am J Respir Cell Mol Biol.

[14] Global Initiative for Chronic Obstructive Lung Disease. http://www.goldcopd.org/guidelines-gold-summary-2011.html. Accessed February 01, 2016.

[15] Folli C, Chiappori A, Pellegrini M, Garelli V, Riccio AM, De Ferrari L (2012). COPD treatment: real life and experimental effects on peripheral NK cells, their receptors expression and their IFN-γ secretion. Pulm Pharmacol Ther.

[16] Davidson TS, DiPaolo RJ, Andersson J, Shevach EM (2007). Cutting Edge: IL-2 is essential for TGF-beta-mediated induction of Foxp3^+^ T regulatory cells. J Immunol.

[17] Zheng SG, Wang J, Wang P, Gray JD, Horwitz DA (2007). IL-2 is essential for TFG-beta to convert naïve CD4^+^CD25^-^ cells to CD25^+^Foxp3^+^ regulatory T cells and for expansion of these cells. J Immunol.

[18] MacNee W (2005). Pulmonary and systemic oxidant/antioxidant imbalance in chronic obstructive pulmonary disease. Proc Am Thorac Soc.

[19] Tuder RM, Yoshida T, Arap W, Pasqualini R, Petrache I (2006). State of the art. Cellular and molecular mechanisms of alveolar destruction in emphysema: an evolutionary perspective. Proc Am Thorac Soc.

[20] Vandivier RW, Henson PM, Douglas IS (2006). Burying the dead: the impact of failed apoptotic cell removal (efferocytosis) on chronic inflammatory lung disease. Chest.

[21] Kang MJ, Lee CG, Lee JY, Dela Cruz CS, Chen ZJ, Enelow R (2008). Cigarette smoke selectively enhances viral PAMP- and virus-induced pulmonary innate immune and remodeling responses in mice. J Clin Invest.

[22] DeMeo DL, Carey VJ, Chapman HA, Reilly JJ, Ginns LG, Speizer FE (2004). Familiar aggregation of FEF(25-75) and FEF(25-75)/FVC in families with severe, early onset COPD. Thorax.

[23] Feghali-Bostwick CA, Gadgil AS, Otterbein LE, Pilewski JM, Stoner MW, Csizmadia E (2008). Autoantibodies in patients with chronic obstructive pulmonary disease. Am J Respir Crit Care Med.

[24] Steinman L (2006). State of the art. Four easy pieces: interconnections between tissue injury, intermediary metabolism, autoimmunity, and chronic degeneration. Proc Am Thorac Soc.

[25] Bluestone JA, Tang Q (2005). How do CD4^+^CD25^+^ regulatory T cells control autoimmunity?. Curr Opin Immunol.

[26] Long SA, Buckner JH (2011). CD4^+^FOXP3^+^ T regulatory cells in human autoimmunity: more than a numbers game. J Immunol.

[27] Lee SH, Goswami S, Grudo A, Song LZ, Bandi V, Goodnight-White S (2007). Antielastin autoimmunity in tobacco smoking-induces emphysema. Nat Med.

[28] Domagala-Kulawik J, Hoser G, Dabrowska M, Safianowska A, Chazan R (2011). CD4^+^/CD25^+^ cells in systemic inflammation in COPD. Scan J Immunol.

[29] Yang L, Ma QL, Yao W, Zhang Q, Chen HP, Wang GS (2011). Relationship between the anti-inflammatory properties of salmeterol/fluticasone and the expression of CD4^+^CD25^+^Foxp3^+^ regulatory T cells in COPD. Respir Res.

[30] Vargas-Rojas MI, Ramírez-Venegas A, Limón-Camacho L, Ochoa L, Hernández-Zenteno R, Sansores RH (2011). Increase of Th17 cells in peripheral blood of patients with chronic obstructive pulmonary disease. Respir Med.

[31] Gregg R, Smith CM, Clark FJ, Dunnion D, Khan N, Chakraverty R (2005). The number of human peripheral blood CD4^+^CD25^high^ regulatory T cells increases with age. Clin Exp Immunol.

[32] Van der Geest KS, Abdulahad WH, Tete SM, Lorencetti PG, Horst G, Bos NA (2014). Aging disturbs the balance between effector and regulatory CD4^+^ T cells. Exp Gerontol.

[33] Raynor J, Lages CS, Shehata H, Hildeman DA, Chougnet CA (2012). Homeostasis and function of regulatory T cells in aging. Curr Opin Immunol.

[34] Vadasz Z, Haj T, Kessel A, Toubi E (2013). Age-related autoimmunity. BMC Med.

[35] Eisenstein EM, Williams CB (2009). The T(reg)/Th17 cell balance: a new paradigm for autoimmunity. Pediatr Res.

[36] Profita M, Albano GD, Riccobono L, Di Sano C, Montalbano AM, Gagliardo R (2014). Increased levels of Th17 cells are associated with non-neuronal acetylcholine in COPD patients. Immunobiology.

[37] Crim C, Calverley PM, Anderson JA, Celli B, Ferguson GT, Jenkins C (2009). Pneumonia risk in COPD patients receiving inhaled corticosteroids alone or in combination: TORCH study results. Eur Respir J.

[38] Suissa S, Patenaude V, Lapi F, Ernst P (2013). Inhaled corticosteroids in COPD and the risk of serious pneumonia. Thorax.

[39] Janson C, Larsson K, Lisspers KH, Ställberg B, Stratelis G, Goike H (2013). Pneumonia and pneumonia related mortality in patients with COPD treated with fixed combinations of inhaled corticosteroids and long acting β2 agonist: observational matched cohort study (PATHOS). BMJ.

[40] Latorre M, Novelli F, Vagaggini B, Braido F, Papi A, Sanduzzi A (2015). Differences in the efficacy and safety among inhaled corticosteroids (ICS)/long-acting beta2-agonists (LABA) combinations in the treatment of chronic obstructive pulmonary disease (COPD): Role of ICS. Pulm Pharmacol Ther.

[41] Blais L, Forget A, Ramachandran S (2010). Relative effectiveness of budesonide/formoterol and fluticasone propionate/salmeterol in a 1-year, population-based, matched cohort study of patients with chronic obstructive pulmonary disease (COPD): Effect on COPD-related exacerbations, emergency department visits and hospitalizations, medication utilization, and treatment adherence. Clin Ther.

[42] Larson T, Gudavalli R, Prater D, Sutton S (2015). Critical analysis of common canister programs: a review of cross-functional considerations and health system economics. Curr Med Res Opin.

[43] Sin DD, Tashkin D, Zhang X, Radner F, Sjöbring U, Thorén A (2009). Budesonide and the risk of pneumonia: a meta-analysis of individual patient data. Lancet.

